# Indirect Effects of Body Dissatisfaction in the Association Between Intolerance of Uncertainty and Disordered Eating Attitudes: A Cross-Sectional Study on Italian University Female Students

**DOI:** 10.3390/jcm14217728

**Published:** 2025-10-30

**Authors:** Giorgia Varallo, Angela Ciaramidaro, Valentina Baldini, Sandro Rubichi, Maristella Scorza

**Affiliations:** 1 Department of Biomedical, Metabolic and Neural Science, University of Modena and Reggio Emilia, Via Campi 287, 41125 Modena, Italy; angela.ciaramidaro@unimore.it (A.C.); valentina.baldini@unimore.it (V.B.); sandro.rubichi@unimore.it (S.R.); maristella.scorza@unimore.it (M.S.); 2 Department of Biomedical and Neuromotor Sciences, University of Bologna, 40126 Bologna, Italy

**Keywords:** clinical psychology, eating behavior, body dissatisfaction, anxiety, university students

## Abstract

**Objectives**: Intolerance of uncertainty (IU) is a transdiagnostic factor implicated in emotional disorders and has recently been linked to maladaptive eating attitudes. Body dissatisfaction, a core risk factor for maladaptive eating, may represent a key pathway through which IU exerts its effects. This study examined whether body dissatisfaction has an indirect effect on the association between IU and disordered eating attitudes in female university students, controlling for body mass index (BMI) and trait anxiety. **Methods**: A cross-sectional study was conducted with 141 female psychology students aged 18–35 years (M = 21.23, SD = 2.31). Participants completed self-report measures of IU (Intolerance of Uncertainty Scale–Short Form), body dissatisfaction (Body Shape Questionnaire), disordered eating attitudes (Eating Attitudes Test-26), trait anxiety (State–Trait Anxiety Inventory), and reported weight and height to calculate BMI. Indirect effects were tested using bootstrapped mediation models. **Results**: IU was positively associated with body dissatisfaction (β = 1.139, *p* = 0.001), which in turn significantly predicted dieting (β = 0.126, *p* < 0.001) and bulimia/food preoccupation (β = 0.033, *p* < 0.001), but not oral control. Bootstrapped analyses showed significant indirect effects of IU on dieting (β = 0.144, 95% CI [0.047, 0.251]) and bulimia/food preoccupation (β = 0.037, 95% CI [0.010, 0.074]) via body dissatisfaction. Direct effects of IU on eating attitudes were not significant. **Conclusions**: IU to be associated with disordered eating attitudes primarily through body dissatisfaction, independently of BMI and anxiety. These findings extend evidence of IU as a cognitive vulnerability for eating-related psychopathology to non-clinical populations, highlighting the need for preventive interventions addressing both body image concerns and IU in female university students.

## 1. Introduction

Intolerance of uncertainty (IU) has been identified as a transdiagnostic process implicated in several psychological disorders, such as anxiety, depression, and obsessive–compulsive disorder [1]. Originally conceptualized within cognitive models of anxiety [1,2,3], IU refers to a dispositional incapacity to tolerate the aversive response triggered by perceived uncertainty, ambiguity, or unpredictability, regardless of the actual likelihood of threat [1]. Individuals reporting high levels of IU typically experience higher anxiety and negative affect when facing ambiguous or unpredictable situations [4] and tend to appraise uncertain situations as inherently threatening, which promotes maladaptive emotion regulation strategies such as hypervigilance, avoidance behaviors, and excessive worry [5,6].

Since its original conceptualization as a key feature of generalized anxiety disorder [1], IU has been increasingly recognized as a transdiagnostic construct underlying a wide spectrum of emotional disorders [6,7,8]. Specifically, since anxiety and eating disorders (EDs) are often comorbid, preliminary evidence has recently started to accumulate regarding the role of IU in EDs [7,8,9,10,11]. Elevated levels of IU have consistently been documented in individuals with EDs [12]. Specifically, higher IU has been found to correlate positively with alexithymia, harm avoidance, depressive symptoms, drive for thinness, and body dissatisfaction [10,11]. In heterogeneous samples of individuals with EDs, IU has also been linked to dietary restraint, purging behaviors, and uncertainty-related concerns specifically tied to body weight, shape, and food, as well as the use of safety behaviors aimed at reducing distress [13,14]. Notably, these associations extend beyond clinical populations; even among non-clinical samples, elevated IU has been related to problematic eating attitudes [15]. It can be hypothesized, as suggested by Bijsterbosch and colleagues [16], that individuals with high IU may perceive the fluctuations of internal bodily related states—such as hunger, satiety, and interoceptive sensations—as well as changes in body weight and shape as aversive, unpredictable, or difficult to interpret, leading to body dissatisfaction and in turn to rigid control over eating and appearance as a maladaptive strategy to reduce perceived unpredictability.

Indeed, body dissatisfaction is considered a core psychopathological feature and one of the strongest predictors of the onset and maintenance of maladaptive eating behaviors [17,18]. Body dissatisfaction refers to the subjective dissatisfaction with body size and/or shape [19,20] and has been strongly associated with maladaptive eating patterns such as restrictive dieting, binge–purge behaviors, and overvaluation of weight and shape [18,21,22,23,24]. Evidence suggests that IU may exacerbate these concerns; for example, a previous study has found a significant association between IU and body dissatisfaction [25], suggesting that individuals with elevated IU might be less able to tolerate appearance-related uncertainties, which may lead to higher body dissatisfaction. Most of the available evidence derives from clinical samples with diagnosed eating disorders. However, the presence of IU and body dissatisfaction in non-clinical populations such as University students is equally relevant. Indeed, the health of university students is a growing public health concern, as this developmental stage is characterized by increased vulnerability to psychological and behavioral difficulties [26]. Eating disorders, in particular, pose a significant risk to both physical and mental health. Recent evidence indicates a marked rise in eating disorder risk among college populations. In a large national study including more than 260,000 U.S. undergraduate students, the prevalence of eating disorder risk increased from 15% in 2013 to 28% in 2020/2021, with the most pronounced increases observed among young women [27]. Also, especially in female University students, body dissatisfaction represents a significant concern due to its prevalence and role in the development of maladaptive eating behaviors [28,29]. This trend is alarming, given the well-documented adverse health outcomes associated with disordered eating [30,31] and underscores the urgent need for expanded prevention and intervention strategies targeting student populations, especially young adult females who appear to be at the greatest risk. Understanding how IU contributes to maladaptive eating attitudes in female university students is crucial for developing preventive strategies. However, to date, no study has investigated the role of IU and body dissatisfaction in maladaptive eating patterns. Thus, we investigated whether body dissatisfaction has an indirect effect on the association between IU and disordered eating attitudes, while also accounting for trait anxiety and BMI. Indeed, these factors might have an impact on the variables included in the model. Higher BMI is associated with a higher level of body dissatisfaction [32,33] and maladaptive eating behaviors [34,35]. Also, anxiety has been shown to play a significant role in IU [36], body dissatisfaction [37] and eating patterns [38,39,40]. We hypothesized that higher levels of IU would be associated with greater disordered eating attitudes, namely (i) dieting behaviors, (ii) bulimia and food preoccupation, (iii) oral control (as measured by Eating Attitudes Test-26), and that body shape concerns would account for indirect effects in these associations, even after adjusting for trait anxiety and BMI.

## 2. Materials and Methods

### 2.1. Participants and Procedure

This study employed a cross-sectional design. The sample consisted of N = 141 female university students, aged between 18 and 35 years, with a mean age of 21.23 ± 2.31. A convenience sampling strategy was adopted. Participants were recruited through an email invitation sent to female students enrolled in the Psychology program. To be included in the study, individuals had to be (i) aged >18 and <35 years, (ii) of female sex assigned at birth, and (iii) fluent Italian speakers. Exclusion criteria were (i) a self-reported current diagnosis of a severe psychiatric disorder (such as psychosis or bipolar disorder), or (ii) a self-reported neurological condition that could interfere with psychological assessment.

Data collection was carried out between 30 September 2024 and February 2025 using Google Forms. All responses were completely anonymous; the anonymity was guaranteed by using Self-Generated Identification Codes. Data were stored in a password-protected file to guarantee confidentiality and data protection. Before beginning the survey, participants read the study information and provided informed consent electronically. All students who accessed the survey link voluntarily completed the entire set of questionnaires, as the platform required completion of all items before submission. Therefore, full data were available for all participants included in the study. At the end of the survey, participants received recognition of one academic credit as compensation for their time and effort. Data collection adhered to the principles of the Declaration of Helsinki and was approved by the University of Modena and Reggio Emilia Ethics Committee (9 August 2024 Prot. 234034). 

### 2.2. Measures

#### 2.2.1. Predictor

##### Intolerance of Uncertainty Scale (IUS)

The Intolerance of Uncertainty Scale—short version, in its Italian validation [41,42] was administered to assess the individual’s tendency to perceive uncertain situations as stressful and threatening. The questionnaire consists of 12 items, rated on a 5-point Likert scale ranging from 1 to 5. Higher scores indicate greater IU. The IUS has demonstrated good internal consistency in the current sample (Cronbach’s α = 0.92).

#### 2.2.2. Indirect Effect Variable

##### Body Shape Questionnaire (BSQ)

Concerns related to body shape were measured using the Italian validation of the Body Shape Questionnaire [43,44]. The BSQ is a 34-item self-report scale designed to assess body dissatisfaction and preoccupation with weight and shape. Participants respond on a 6-point Likert scale, ranging from 1 to 6, referring to their experiences over the past four weeks. Higher scores reflect greater body dissatisfaction and concerns about physical appearance. The reliability indices in the current sample are excellent (Cronbach’s α = 0.94).

#### 2.2.3. Outcomes

##### Eating Attitudes Test-26 (EAT-26)

Disordered eating attitudes and behaviors were assessed with the Italian validation of the Eating Attitudes Test-26 [45,46]. The EAT-26 is a widely used screening tool for identifying the risk of eating disorders in community and clinical samples. It includes 26 items rated on a 6-point Likert scale ranging from always to never. The total score is considered in this study as a global index of disordered eating symptomatology, with higher values indicating greater severity. A cut-off score of 20 is generally used to identify individuals at risk for an eating disorder. The EAT-26 is composed of three subscales. The Dieting subscale assesses restrictive eating behaviors, avoiding high-calorie foods, and preoccupation with thinness (e.g., “I am terrified about being overweight”). The Bulimia and Food Preoccupation subscale measures tendencies toward binge eating, purging, and intrusive food-related thoughts (e.g., “I have gone on eating binges where I feel that I may not be able to stop”). The Oral Control subscale evaluates the level of self-control over eating and perceived social pressure to eat more or less (e.g., “I feel that others would prefer if I ate more”). Higher scores indicate greater severity of eating-related concerns, with a total score of 20 or above commonly used as a clinical cut-off for identifying individuals at risk for an eating disorder. The current sample reported satisfactory internal consistency (Cronbach’s α = 0.90).

#### 2.2.4. Confounders

##### Body Mass Index (BMI)

Body Mass Index was calculated as weight in kilograms divided by the square of height in meters (kg/m^2^), based on self-reported values provided by participants. BMI was treated as a continuous variable in all analyses.

##### State–Trait Anxiety Inventory (STAI)

The State–Trait Anxiety Inventory—Trait subscale [47,48] is a widely used self-report measure designed to assess relatively stable individual differences in anxiety proneness, that is, the general tendency to perceive situations as threatening and to respond with increased anxiety. The trait subscale consists of 20 items rated on a 4-point Likert scale (from almost never to almost always), with higher scores indicating greater levels of dispositional anxiety. In the current sample, the internal consistency was good (Cronbach’s α = 0.84).

### 2.3. Statistical Analysis

Analyses were performed using JASP (version 0.14 [49]). There were no missing data, as responses to all questionnaire items were mandatory. Participants could complete and submit the survey only if all items had been answered. As a first step, we examined the descriptive statistics of the variables of interest. For each measure, we reported mean values, standard deviations, and ranges.

We then explored the univariate associations among the main variables through Pearson correlations. Thus, we tested the indirect effect of body dissatisfaction (i.e., BSQ total score) in the association between IU (i.e., IUS total score) and three outcomes: (i) dieting behaviors, (ii) bulimia, and food preoccupation, and (iii) oral control subscale. Trait anxiety and BMI were included as covariates to account for their potential influence on these associations.

Indirect effect associations were assessed by estimating the direct, indirect, and total effects. Indirect effects were tested using a percentile-bootstrapping procedure with 5000 resamples [50,51]. For each path, we reported standardized coefficients together with standard errors, z-values, *p*-values, and 95% confidence intervals. All analyses were two-tailed, and a probability value below 0.05 was considered statistically significant.

## 3. Results

### 3.1. Descriptive Statistics and Correlations

Results of descriptive statistics are reported in Table 1.

The variable Age showed high skewness and kurtosis values, indicating a non-normal distribution; however, this pattern is consistent with the restricted range of the sample (with most students aged between 18 and 23 years and very few in the upper range of 30–35 years). However, Pearson correlations are considered robust to violations of normality. Bivariate correlations among study variables indicated that IUS was significantly and positively correlated with body shape concerns (BSQ; r = 0.376, *p* < 0.001), dieting (r = 0.348, *p* < 0.001), bulimia and food preoccupation (r = 0.320, *p* < 0.001), oral control (r = 0.224, *p* < 0.01), BMI (r = 0.194, *p* < 0.05), and trait anxiety (STAI; r = 0.237, *p* < 0.01). BSQ showed the strongest associations, being highly correlated with dieting (r = 0.752, *p* < 0.001), bulimia and food preoccupation (r = 0.558, *p* < 0.001), and BMI (r = 0.523, *p* < 0.001), and moderately with STAI (r = 0.329, *p* < 0.001). BMI was positively related to dieting (r = 0.343, *p* < 0.001) and bulimia and food preoccupation (r = 0.351, *p* < 0.001), but negatively to oral control (r = –0.180, *p* < 0.05). STAI was moderately associated with dieting (r = 0.247, *p* < 0.01), but showed no significant relation with BMI (r = 0.029, ns), bulimia and food preoccupation (r = 0.120, ns), or oral control (r = 0.144, ns). Finally, BSQ and oral control were not significantly correlated (r = 0.140, ns).

### 3.2. Indirect Effect Analysis

**Figure 1:**
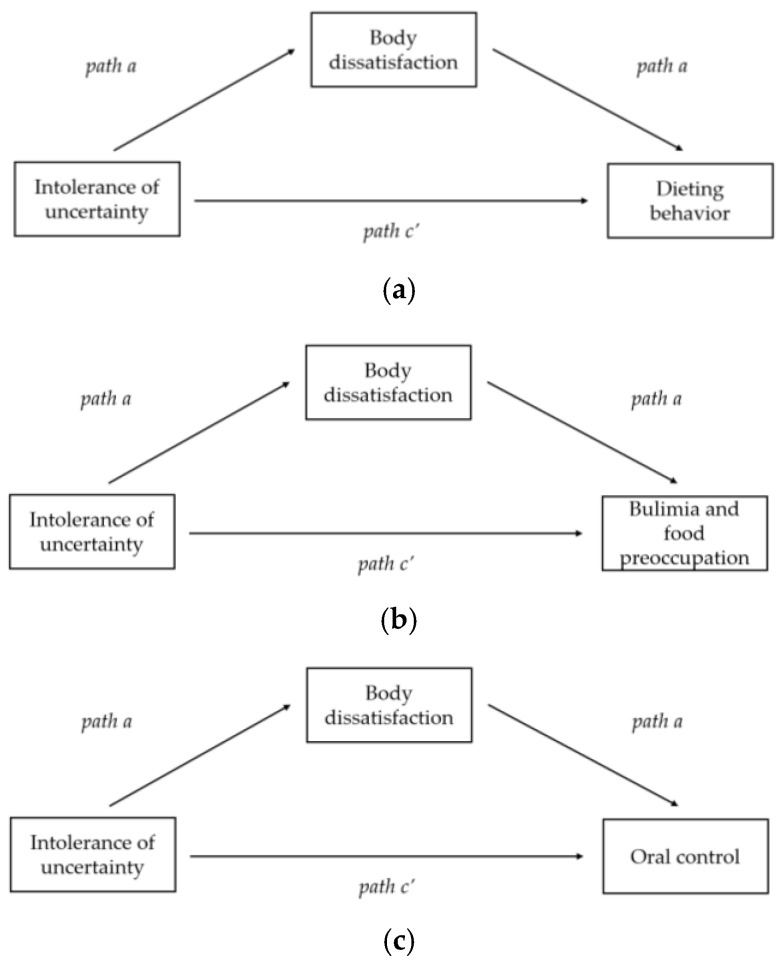
Mediation models illustrating the indirect effects of intolerance of uncertainty on maladaptive eating patterns through body dissatisfaction: (**a**) model with dieting behavior as the outcome; (**b**) model with bulimia and food preoccupation as the outcome; (**c**) model with oral control as the outcome. All models were adjusted for BMI and trait anxiety levels.

The hypothesized model included IU (i.e., IUS total score) as a predictor, body shape concerns (i.e., BSQ total score) as the indirect effect variable, and three outcomes: dieting behaviors, bulimia and food preoccupation, and oral control subscale. Trait anxiety (i.e., STAI total score) and BMI were also modeled as covariates (Figure 1).

As hypothesized, IUS was positively associated with body shape concerns (BSQ total score; β = 1.139, SE = 0.352, z = 3.239, *p* = 0.001). In turn, BSQ significantly predicted both the Dieting subscale (β = 0.126, SE = 0.018, z = 7.196, *p* < 0.001) and the Bulimia and Food Preoccupation subscale (β = 0.033, SE = 0.007, z = 4.403, *p* < 0.001). The association between BSQ and Oral Control was positive but did not reach statistical significance (β = 0.014, *p* = 0.078) (Table 2).

Bootstrapped indirect effects indicated that IUS was indirectly associated with Dieting through BSQ (β = 0.144, SE = 0.043, z = 3.326, *p* < 0.001, 95% CI [0.047, 0.251]) and with Bulimia and Food Preoccupation through BSQ (β = 0.037, SE = 0.013, z = 2.971, *p* = 0.003, 95% CI [0.010, 0.074]). The indirect effect of IUS on Oral Control via BSQ was non-significant (β = 0.016, *p* = 0.105).

Direct effects of IUS on the EAT-26 subscales were not significant (Dieting: β = 0.066, *p* = 0.236; Bulimia and Food Preoccupation: β = 0.038, *p* = 0.116; Oral Control: β = 0.070, *p* = 0.074). The total effects were significant for Dieting (β = 0.209, SE = 0.060, z = 3.462, *p* < 0.001, 95% CI [0.064, 0.358]) and Bulimia and Food Preoccupation (β = 0.075, SE = 0.024, z = 3.120, *p* = 0.002, 95% CI [0.017, 0.134]), and also for Oral Control (β = 0.086, SE = 0.029, z = 3.006, *p* = 0.003, 95% CI [0.013, 0.161]).

Regarding covariates, trait anxiety (i.e., STAI) was positively associated with both IUS (β = 0.461, SE = 0.169, z = 2.738, *p* = 0.006) and BSQ (β = 2.315, SE = 0.696, z = 3.325, *p* < 0.001), indicating that higher anxiety contributes to greater IU and body dissatisfaction. BMI was associated with both IUS (β = 0.516, SE = 0.246, z = 2.094, *p* = 0.036) and BSQ (β = 5.930, SE = 0.989, z = 5.997, *p* < 0.001). Interestingly, BMI was negatively associated with Oral Control (β = –0.296, SE = 0.115, z = –2.582, *p* = 0.010), suggesting that higher BMI is linked to reduced perceptions of control around eating. Neither STAI nor BMI showed significant direct associations with the Dieting or Bulimia and Food Preoccupation subscales. All paths are reported in Table S1.

## 4. Discussion

In this study, we examined how IU relates to disordered eating attitudes and BMI through body shape concerns in a sample of female University students. In summary, our results showed that IU did not exert direct effects on either disordered eating attitudes (i.e., EAT score) BMI. Instead, significant indirect effects emerged through body shape concerns (i.e., BSQ score), in the association between IU and Dieting behaviors and Bulimia and food preoccupation, adjusting for trait-anxiety levels.

Consistent with our main hypothesis, IU was indirectly associated with disordered eating attitudes, specifically the Dieting and Bulimia and Food Preoccupation subscales of the EAT-26, through the indirect role of body dissatisfaction. This finding suggests that individuals with higher levels of IU may be more prone to experience heightened concerns regarding body shape, which in turn contributes to maladaptive eating behaviors such as restrictive eating patterns and bulimic tendencies. Importantly, these indirect effects emerged even after adjusting for trait anxiety and BMI.

Our results support the notion that IU may represent a cognitive vulnerability also in eating-related disorders [12,52] since it might exert its influence largely through body dissatisfaction. These findings corroborate the notion that IU serves as a transdiagnostic vulnerability factor for eating-related psychopathology [7], extending the existing evidence to non-clinical populations. Available evidence highlighted that individuals with EDs reported higher levels of IU compared to healthy controls [11,53]. A study conducted on a non-clinical sample of university students with problematic eating attitudes, as assessed with the EAT-26, reported similar findings, showing that individuals with problematic eating attitudes exhibited higher levels of IU [15]. In our study, however, IU was not directly associated with any of the disordered eating attitudes evaluated. This result is not in line with previous evidence, in which IU was found to be directly associated with restraint [13], a construct comparable to dieting. However, this latter study was conducted on female individuals with a diagnosis of eating disorders and assessed eating attitudes using the Eating Disorder Examination Questionnaire, and these differences might explain these discrepancies.

IU has recently been postulated as a potential underlying mechanism involved in the maintenance of body dissatisfaction [25,54]. Although the number of studies examining the association between IU and body dissatisfaction remains limited, existing evidence indicates, indeed, a significant relationship between the two constructs [11,16,25]. Interestingly, a study conducted by Cuesta-Zamora [54] on female University students, found that experimental manipulation of IU-states led to a reduction in body shape concerns. Their findings showed that participants from the Reducing IU Condition perceived themselves as significantly less concerned about their body shape following the experimental task, even when controlling for baseline levels of body dissatisfaction, IU, and exercise weight control attitudes. Our results are consistent with this evidence, highlighting the role of IU on body dissatisfaction and further extending these findings to a non-clinical population of Italian female university students.

To the best of our knowledge, our study is the first to examine the indirect association of IU on disordered eating attitudes via body dissatisfaction. The indirect role of body shape concerns is pivotal. Prior work has linked body dissatisfaction to eating-related psychopathology [18,23]; however, our findings build on that evidence by showing that body dissatisfaction might serve as a proximal factor connecting IU to dieting and bulimia tendencies. Our results are similar to those of Renjan and colleagues [7], who investigated IU within a clinical eating-disorder sample. They found significant indirect effects of IU on disordered eating attitudes such as dietary restraint and purging via overvaluation of eating, weight, and shape. However, in this study, the sample was clinical, and the model did not account for levels of anxiety and BMI. Taken together, these results are in line with cognitive-behavioral models of eating disorders that emphasize the central role of body dissatisfaction [17,18] in the maintenance of disordered eating.

An additional point concerns the lack of indirect or direct effects on the Oral Control subscale. In our sample of non-clinical university students, Oral Control scores were generally very low and showed limited variability, suggesting a possible floor effect that may have reduced the likelihood of detecting significant associations. Moreover, the construct underlying Oral Control may be more strongly influenced by cultural and familial norms (e.g., perceptions that “others would prefer me to eat more or less”) rather than by cognitive processes such as IU.

Finally, several psychological factors show an association with eating disordered attitudes, such as psychological inflexibility [55,56] and emotion dysregulation [56,57,58]. Future research might explore the combined interaction of these factors with IU on eating disordered attitudes.

### 4.1. Limitations

Several limitations should be mentioned. The cross-sectional design does not establish temporal precedence; longitudinal and experimental studies are needed to test the proposed pathway from IU to body shape concerns to eating-related outcomes. Generalization of the present findings should be made with caution. The sample comprised a single-site cohort of female psychology students aged 18–35 years, recruited in Italy. Thus, the results may not extend to males, gender-diverse individuals, older or younger age groups, non-student samples, or clinical populations with diagnosed eating disorders. Finally, all measures relied exclusively on self-report instruments, which may be subject to response biases such as social desirability.

### 4.2. Clinical Implications

The findings of this study suggest several implications for university-based psychological support services. Building on the recommendations of Sternheim et al. [59] in clinical populations, addressing anxiety maintenance processes—such as IU—may represent a valuable target in preventive programs aimed at reducing body shape concerns and mitigating the risk of body image and eating disorders, also in non-clinical populations. However, as the mediation observed in this study was statistical, intervening on body dissatisfaction or IU alone may be suboptimal from a clinical perspective. It is therefore advisable to simultaneously address both IU and body image concerns to maximize efficacy. Specifically, interventions designed to reduce body dissatisfaction—such as those focused on decreasing overvaluation of weight and shape, reducing body checking and avoidance behaviors, employing mirror exposure, and promoting cognitive restructuring—may be more effective when they also enhance individuals’ ability to tolerate uncertainty.

## Figures and Tables

**Table 1:** Descriptive statistics of the total sample (N = 141).

Variable	M (SD)	Min	Max	Skewness	Kurtosis
Age (in years)	21.23 (2.31)	18.00	35.00	3.43	10.42
EAT-26 total score	12.41 (11.37)	3.00	55.00	1.90	2.60
Dieting behavior subscale	6.04 (7.47)	0	35	1.89	2.47
Bulimia and food preoccupation subscale	4.44 (2.87)	0	15	1.86	2.16
Oral control over eating subscale	1.94 (3.276)	0	18	2.21	2.04
IUS	34.53 (10.30)	15.00	59.00	0.21	−0.55
BSQ	95.31 (46.82)	34.00	189.00	0.42	−1.16
BMI	21.31 (3.74)	15.42	37.46	1.56	2.93
STAI	47.65 (5.24)	34	60	−0.17	−0.23

EAT-26 = Eating Attitudes Test; IUS = Intolerance of Uncertainty Scale; BSQ = Body Shape Questionnaire; BMI = Body Mass Index; STAI = State Trait Anxiety Inventory.

**Table 2:** Results of indirect analysis of intolerance of uncertainty on EAT-26 subscales through body dissatisfaction.

					95% Confidence Interval	
	β	SE	z-Value	*p*	Lower	Upper
*Direct effects—path c’*						
IU on Dieting	0.066	0.055	1.185	0.236	−0.044	0.174
IUS on Bulimia and food preoccupation	0.038	0.024	1.572	0.116	−0.009	0.084
IU on Oral control	0.070	0.039	1.787	0.074	−0.007	0.147
*Indirect effect—path ab*						
IU on Dieting via BSQ	0.144	0.043	3.326	<0.001	0.047	0.251
IU on Bulimia and food control via BSQ	0.037	0.013	2.971	0.003	0.010	0.074
IU on Oral control via BSQ	0.016	0.010	1.622	0.105	−0.002	0.043
*Total effect—path c*						
IU on Dieting	0.209	0.060	3.462	<0.001	0.064	0.358
IU on Bulimia and food preoccupation	0.075	0.024	3.120	0.002	0.017	0.134
IU on Oral control	0.086	0.029	3.006	0.003	0.013	0.161

Note: IU = Intolerance of Uncertainty (IUS-12, Intolerance of Uncertainty Scale—Short Form); BSQ = Body Shape Questionnaire; BMI = Body Mass Index.

## Data Availability

Data are available from the corresponding author.

## Supplementary Materials

The following supporting information can be downloaded at: https://www.mdpi.com/article/10.3390/jcm14217728/s1, Table S1: Path coefficients.
