# Supplementary material for: Indirect Effects of Body Dissatisfaction in the Association Between Intolerance of Uncertainty and Disordered Eating Attitudes: A Cross-Sectional Study on Italian University Female Students
Source: J Clin Med. 2025 Oct 30;14(21):7728. doi: 10.3390/jcm14217728 (PMC12609605; doi:10.3390/jcm14217728)
Supplement: Supplementary file 1 [file jcm-14-07728-s001.zip › jcm-3918712-supplementary.pdf]

Table S1. Path coefficients.

			B	SE	Z-value	P	95% Confidence Interval	
							Lower	Upper
Body dissatisfaction	→	Dieting	0.126	0.018	7.196	< .001	0.091	0.159
Intolerance of uncertainty	→	Dieting	0.066	0.055	1.185	0.236	-0.044	0.174
Body dissatisfaction	→	Bulimia and food preoccupation	0.033	0.007	4.403	< .001	0.018	0.047
Intolerance of uncertainty	→	Bulimia and food preoccupation	0.038	0.024	1.572	0.116	-0.009	0.084
Body dissatisfaction	→	Oral control	0.014	0.008	1.764	0.078	-0.002	0.030
Intolerance of uncertainty	→	Oral control	0.070	0.039	1.787	0.074	-0.007	0.147
Intolerance of uncertainty	→	Body dissatisfaction	1.139	0.352	3.239	0.001	0.410	1.794
Trai anxiety	→	Intolerance of uncertainty	0.461	0.169	2.738	0.006	0.113	0.779
BMI	→	Intolerance of uncertainty	0.516	0.246	2.094	0.036	0.035	1.001
Trai anxiety	→	Body dissatisfaction	2.315	0.696	3.325	< .001	0.931	3.683
BMI	→	Body dissatisfaction	5.930	0.989	5.997	< .001	4.149	8.048
Trai anxiety	→	Dieting	-0.025	0.091	-0.270	0.788	-0.205	0.152
BMI	→	Dieting	-0.162	0.158	-1.030	0.303	-0.463	0.156
Trai anxiety	→	Bulimia and food preoccupation	-0.055	0.051	-1.088	0.276	-0.152	0.044
BMI	→	Bulimia and food preoccupation	0.043	0.091	0.472	0.637	-0.165	0.192
Trai anxiety	→	Oral control	0.035	0.061	0.569	0.570	-0.082	0.159
BMI	→	Oral control	-0.296	0.115	-2.582	0.010	-0.560	-0.107

Note: BMI= Body Mass Index.
